# Advances in Methylmercury Toxicology and Risk Assessment

**DOI:** 10.3390/toxics7020020

**Published:** 2019-04-01

**Authors:** Hing Man Chan

**Affiliations:** Department of Biology, University of Ottawa, 30 Marie Curie, Ottawa, ON K1N 6N5, Canada; laurie.chan@uottawa.ca; Tel.: +613-562-5800 (ext. 7116)

Mercury (Hg) is a global pollutant that affects the health of both humans and ecosystems. Wildlife and humans are exposed to Hg primarily through diet in the form of methylmercury (MeHg) because MeHg bioaccumulates and biomagnifies in the food web. Among Hg species, MeHg toxicity is significantly more damaging to the nervous system in early developmental stages because it leads to alterations in both structure and function. Even at low doses, prenatal exposure to MeHg can disrupt fetal brain development leading to long-term effects even in adulthood. 

This Special Issue collected three review papers and six research articles that report the latest findings on the mechanisms of MeHg toxicology and its impacts on environmental health. Despite well-described neurobehavioral effects, the mechanisms of MeHg-induced toxicity are not completely understood. Antunes dos Santos et al. [1] reviewed the latest evidence indicating oxidative stress to be an important molecular mechanism in MeHg-induced intoxication. They have also highlighted the role of the PI3K/Akt signalling pathway and the nuclear transcription factor NF-E2-related factor 2 (Nrf2) in MeHg-induced redox imbalance. These results are important for future research in identifying the adverse outcome pathway for MeHg. In addition to its neurotoxicity, MeHg exposure has also been reported to affect heart health; however, evidence remains inconsistent. Karita et al. [2] reviewed the latest evidence on the association between MeHg and heart rate variability (HRV). They identified 13 studies examining the effect of MeHg exposure on HRV in human populations in the Faroe Islands, Seychelles, and other countries. They found that eight studies showed significant associations and five studies did not show any significant association. They concluded that the effects of MeHg might differ for prenatal and postnatal exposures and the HRV parameters calculated by frequency domain analysis were more sensitive than those by time domain analysis. These results suggest that HRV should be included in the risk characterization of MeHg. The third review by Sakamoto et al. [3] reported valuable lessons learned from the MeHg pollution in Minamata and the significance is discussed below. 

Takahashi et al. [4] investigated the relationship between a chemokine, C-C motif Chemokine Ligand 4 (CCL4) expression and MeHg toxicity. Using both in vivo and in vitro approaches, they found that induction of CCL4 expression occurred prior to cytotoxicity caused by MeHg. They also showed that CCL4 is a protective factor against MeHg toxicity. Lee et al. [5] investigated the roles of a metal-binding protein metallothionein-III (MT-III) in chemokine gene expression changes in response to MeHg and Hg mercury vapour in the cerebrum and cerebellum in wild-type and MT-III knockout mice. They showed that the expression of Ccl12 and Cxcl12 was increased in the cerebrum by MeHg exposure in the wild-type mice only. Their results confirmed that MT-III plays a role in the expression of some chemokine genes in response to MeHg. The new understanding of these two studies on the protective roles of chemokines against the MeHg toxicity in the brain is important for the future development of treatments. 

The mechanisms responsible for MeHg-induced changes in adult neuronal function, when exposure occurs primarily during fetal development, are not yet understood. Yuan et al. [6] dosed primary cortical precursor cells obtained from mice embryo with MeHg and found the cortical precursor exposed to extremely low dose (0.25 nM) of MeHg increased neuronal differentiation; while its proliferation was inhibited. In comparison, reduced neuronal differentiation was observed in the higher dose groups. These results suggest that sub-nanomolar MeHg exposure may deplete the pool of neural precursors by increasing premature neuronal differentiation. This can lead to long-term neurological effects in adulthood as neural precursors are important for adult neurogenesis. In contrast, the higher MeHg doses cause more immediate toxicity during infant development leading to neurobehaviour effects observed in infants and children. These results may explain the long delay effects of MeHg reported in Minamata, Japan as reported by Takaoka et al. [7]. In 2009, Takaoka et al. performed health surveys on 973 residents in the polluted area and 142 residents from a control area. Their results show that Minamata disease had spread outside of the central area and could still be observed almost 50 years after the Chisso Company’s factory halted the dumping of Hg-polluted waste water in 1968. Results presented in this paper provide invaluable information on the long-term effects of MeHg, particularly on the wide range of clinical symptoms observed in adults who had no reported symptoms at younger age. Sakamoto et al. [3] reported a comprehensive review of the exposure and health effects which result from the MeHg pollution in Minamata. Information acquired during this review included the use of preserved umbilical cords to study the extent of pre-natal exposure, the higher sensitivity of male than female newborns to MeHg, and the neuropathology and kinetics of MeHg in fetuses. The sex-specific results were also confirmed by the results of a cohort study conducted by Tatsuta et al. [8]. To clarify the effects of MeHg on child development, a longitudinal prospective birth cohort study was conducted by the Tohoku Study of Child Development (TSCD) in Japan. Tatsuta et al. [8] reported the latest findings of the TSCD, showing that prenatal MeHg exposure affected psychomotor development in 18-month-olds, especially in males. The results of these studies provide important new information on the comparative use of different biomarkers, such as maternal blood or hair, for the dose-response assessment for MeHg on fetuses and child development.

Many toxic trace metals including Hg have been shown to induce immunotoxicity. However, there is a lack of specific biomarkers in peripheral blood samples for the effects of different chemical exposure. Monastero et al. [9] conducted a pilot study to correlate the blood levels of three metals, including cadmium, lead and mercury, with differences in the expression of 98 genes associated with stress, toxicity, inflammation, and autoimmunity in 24 participants from the Long Island Study of Seafood Consumption. They found that the expression of three genes (*IL1RAP*, *CXCR1*, and *ITGB2*) was negatively associated with Hg. This preliminary result suggests the possibility of using gene expression in blood samples to study the effects of MeHg on immune functions in future population studies. 

In summary, this collection of papers on the toxicology and risk assessment provides useful new information on the mechanisms of MeHg toxicity and methods of improving risk assessment of MeHg. 
